# 1907. Initiation of Comfort Measures Only Care in the Medical Intensive Care Unit during the COVID-19 Pandemic

**DOI:** 10.1093/ofid/ofac492.1534

**Published:** 2022-12-15

**Authors:** Max Jacobs, Helene Rovnan, Deeksha Ramanujam, Amina Hamza, Stephen Caucci, Tariq Cheema

**Affiliations:** Allegheny Health Network, Pittsburgh, Pennsylvania; Allegheny Health Network, Pittsburgh, Pennsylvania; Allegheny Health Network, Pittsburgh, Pennsylvania; Allegheny Health Network, Pittsburgh, Pennsylvania; Lake Erie College of Osteopathic Medicine, Pittsburgh, Pennsylvania; Allegheny Health Network, Pittsburgh, Pennsylvania

## Abstract

**Background:**

The COVID-19 pandemic brought an unprecedented disruption to many hospital standard operating procedures including changing family visitation policies. As an infection control policy, most hospitals denied or limited visitors and patient families were updated via phone or video conferencing. There are several plausible reasons to suspect that altered communication techniques and heightened public awareness of critical illness during the pandemic impacted end of life care and decision making.

**Methods:**

We conducted a retrospective observational cohort study from a tertiary care hospital to determine if more patients in the ICU were transitioned to comfort measures only (CMO) care during COVID than in the same period the year before when COVID had not yet arrived in the United States (“pre-pandemic”). Collected information included demographics (Table 1), reason for ICU, co-morbidities, code status at admission, length of ICU stay, cost of care during ICU admission, and disposition at discharge. All analyses began using the Kolmogorov -Smirnov test. Normally distributed continuous variables are compared between groups using the independent-samples t-test. Non-normally distributed continuous variables are compared between groups using the Mann-Whitney U test.

**Results:**

There were no statistically significant differences in baseline demographic and clinical characteristics of patients admitted to the medical ICU during the pre-pandemic versus pandemic periods (Table 1). Among the 266 patients admitted during the pre-pandemic period, 11.7% (31/266) were discharged with CMO code status. Among the 160 patients admitted in the pandemic period, 8.8% (14/160) were discharged with CMO code status (Table 2). The median APACHE score was significantly higher in the pre-pandemic period ([median=18 (interquartile range = 11) compared to the pandemic period ([median=15 (interquartile range = 1), (Mann-Whitney U = 17146.50, p = .008). The cost of an ICU admission during the pandemic period increased 0.099%.

**Table 1:**
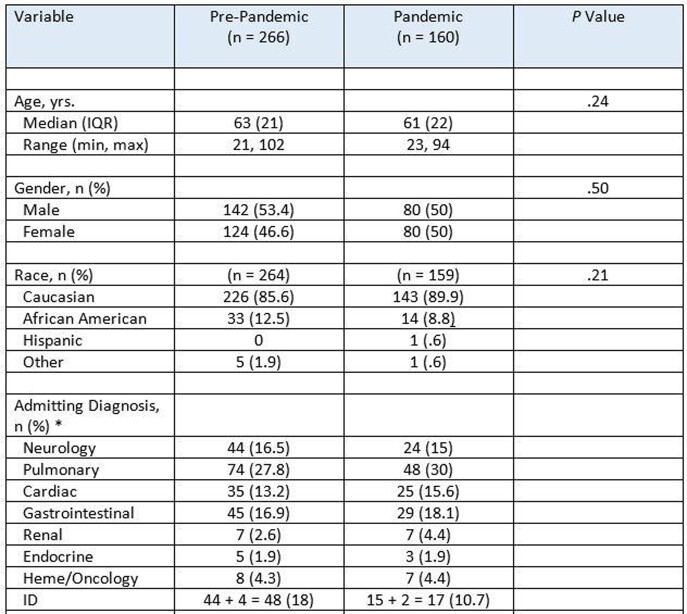
Baseline demographic and clinical characteristics of patients admitted to the medical ICU pre- versus post-pandemic (n = 426).

**Table 2:**
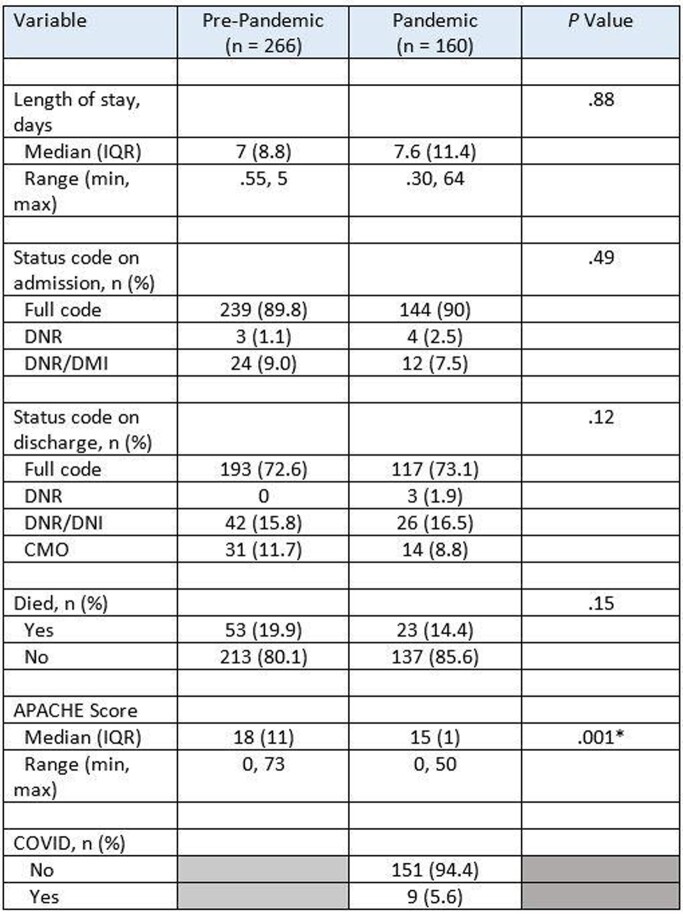
Discharge status code and outcomes of patients admitted to the medical ICU in the pre- versus post-pandemic (n = 426).

**Conclusion:**

This study shows that despite the highmorbidity and mortality associated with COVID, during the study time periods, the limitation of in-person visitors did not have an impact on the frequency of patients transitioned to CMO or decreased hospital expenditure.

**Disclosures:**

**Tariq Cheema, MD**, GSK,BI,ASTRA ZENECA,Regenoron: Honoraria.

